# The Effects of 10 Hz Transcranial Alternating Current Stimulation on Audiovisual Task Switching

**DOI:** 10.3389/fnins.2018.00067

**Published:** 2018-02-13

**Authors:** Michael S. Clayton, Nick Yeung, Roi Cohen Kadosh

**Affiliations:** Department of Experimental Psychology, University of Oxford, Oxford, United Kingdom

**Keywords:** transcranial alternating current stimulation (tACS), Alpha oscillations, task switching, multisensory perception, stabilization

## Abstract

Neural oscillations in the alpha band (7–13 Hz) are commonly associated with disengagement of visual attention. However, recent studies have also associated alpha with processes of attentional control and stability. We addressed this issue in previous experiments by delivering transcranial alternating current stimulation at 10 Hz over posterior cortex during visual tasks (alpha tACS). As this stimulation can induce reliable increases in EEG alpha power, and given that performance on each of our visual tasks was negatively associated with alpha power, we assumed that alpha tACS would reliably impair visual performance. However, alpha tACS was instead found to prevent both deteriorations and improvements in visual performance that otherwise occurred during sham & 50 Hz tACS. Alpha tACS therefore appeared to exert a stabilizing effect on visual attention. This hypothesis was tested in the current, pre-registered experiment by delivering alpha tACS during a task that required rapid switching of attention between motion, color, and auditory subtasks. We assumed that, if alpha tACS stabilizes visual attention, this stimulation should make it harder for people to switch between visual tasks, but should have little influence on transitions between auditory and visual subtasks. However, in contrast to this prediction, we observed no evidence of impairments in visuovisual vs. audiovisual switching during alpha vs. control tACS. Instead, we observed a trend-level reduction in visuoauditory switching accuracy during alpha tACS. *Post-hoc* analyses showed no effects of alpha tACS in response time variability, diffusion model parameters, or on performance of repeat trials. EEG analyses also showed no effects of alpha tACS on endogenous or stimulus-evoked alpha power. We discuss possible explanations for these results, as well as their broader implications for current efforts to study the roles of neural oscillations in cognition using tACS.

## Introduction

Perhaps the most prominent oscillation produced by the human brain is occipitoparietal alpha: a 7–13 Hz rhythm recorded maximally over posterior cortex. Increases in the power of these oscillations are observed during eyes-closed rest (Berger, 1929; Barry et al., 2007) and during periods of reduced attention to visual tasks (O'Connell et al., 2009; Chaumon and Busch, 2014). Alpha oscillations are therefore suggested to reflect disengagement of visual processing (e.g., Foxe and Snyder, 2011). However, recent studies have also reported positive associations between alpha power and many neurocognitive functions that appear critical to visual attention (Clayton et al., 2017).

For example, alpha oscillations have been suggested to facilitate integration of attention-related brain networks (Sadaghiani et al., 2012), and communication of top-down predictions to visual cortex (Sherman et al., 2016). Alpha oscillations may also promote stability in visual processing. This idea is suggested by multistable perception studies showing decreases in alpha power immediately before changes in the perception of ambiguous stimuli (e.g., Necker cubes) (Isoglu-Alka et al., 2000; Strüber and Herrmann, 2002; Piantoni et al., 2017). Such power decreases may reflect destabilization of ongoing, perceptual interpretations (e.g., Strüber and Herrmann, 2002). On a similar note, decreases in alpha power are observed when people switch their attention between different visual tasks (Poljac and Yeung, 2012; Foxe et al., 2014). Consequently, in addition to their links with attentional disengagement, alpha oscillations also exhibit positive associations with the maintenance of ongoing, visual processing.

One method used to investigate the roles of neural oscillations in cognition is transcranial alternating current stimulation (tACS). This procedure involves delivery of sinusoidal, electrical currents to the brain via electrodes positioned on the scalp. These currents can modulate both the power and phase of neural oscillations at the frequency of stimulation (Zaehle et al., 2010; Battleday et al., 2014; although effects at other frequencies have also been observed; Neuling et al., 2012a; Ruhnau et al., 2016). One therefore assumes, if a specific cognitive process is affected by tACS at a specific frequency, that the cognitive process must rely to some extent on neural activity at that frequency (e.g., Thut et al., 2011; Clayton et al., 2015).

In previous experiments conducted by our group, tACS was delivered at 10 Hz over posterior cortex (alpha tACS) while participants performed visual tasks. Poor performance on these tasks was previously associated with increased EEG alpha power (O'Connell et al., 2009; Chaumon and Busch, 2014; Gonzalez-Rosa et al., 2015). Given that alpha tACS has also been reported to increase alpha power (e.g., Helfrich et al., 2014), we therefore predicted that this stimulation would consistently impair visual performance. However, alpha tACS was instead found to prevent changes in performance over an extended period of time (~15 min). Specifically, from the start of stimulation onwards, alpha tACS limited the slope of deteriorations in attention that otherwise occurred during sham and 50 Hz tACS. Furthermore, when participants performed a task where rapid learning was observed, alpha tACS was found to limit the slope of these improvements. Such effects were not observed in an auditory control task, indicating specificity to the visual domain.

A possible explanation for these results was that alpha tACS exerts a stabilizing effect on visual attention. This idea is consistent with previously mentioned evidence suggesting positive associations between alpha power and stability in visual processing (Strüber and Herrmann, 2002; Piantoni et al., 2017). We dedicated the current, pre-registered experiment to investigating this view. We did this by delivering alpha tACS during a task that requires rapid switches of attention between motion, color, and auditory subtasks. As in our previous experiments, the effects of alpha tACS were compared to those of sham and 50 Hz tACS. Our central hypothesis was that, if alpha tACS stabilizes (i.e., prevents changes in) visual attention, this stimulation should make it harder for participants to switch their attention between visual tasks. This view is consistent with previous evidence that effective switching between visual tasks is negatively associated with EEG alpha power (e.g., Poljac and Yeung, 2012). Importantly though, as alpha tACS was previously found to influence visual performance alone, we assumed that alpha tACS would not affect switches between auditory and visual subtasks. Electroencephalography (EEG) data was collected throughout the experiment. We expected to observe significant enhancements in EEG alpha power following alpha tACS, and that such enhancements would be associated with increased visuovisual vs. audiovisual switch costs.

## Materials and methods

### Pre-registration and participants

The hypotheses, method, and planned analyses for this experiment were pre-registered with the Open Science Framework before the data were collected (*The effects of 10 Hz tACS on visual task switching*; https://osf.io/f6b3s/). A total of 40 subjects participated in the study. This sample size was chosen based on the sample sizes of our previous experiments, which observed significant effects of alpha tACS on visual task performance. Previous studies suggest that alpha tACS increases EEG alpha power only when baseline alpha power is low (Neuling et al., 2013; Alagapan et al., 2016). Consequently, to limit the influence of such effects on our data, mean alpha power before the delivery alpha tACS was z-transformed (individual alpha frequency ±2 Hz [defined below]). A single subject exhibited a *z*-score above of 4.09, reflecting a baseline alpha power that was significantly greater than the sample mean (*p* < 0.01). Following the methods of Kasten and Herrmann (2017), this subject was excluded from all analysis. In addition, one subject was excluded because their overall task accuracy was more than 3 standard deviations below the sample mean. Another participant was also excluded because they failed to press any response button on the last block of one session. The final sample therefore consisted of 37 subjects (17 females, 8 left-handed, mean age = 23.64, *SD* = 4.35).

### Electroencephalography and transcranial alternating current stimulation

**Figure 1:**
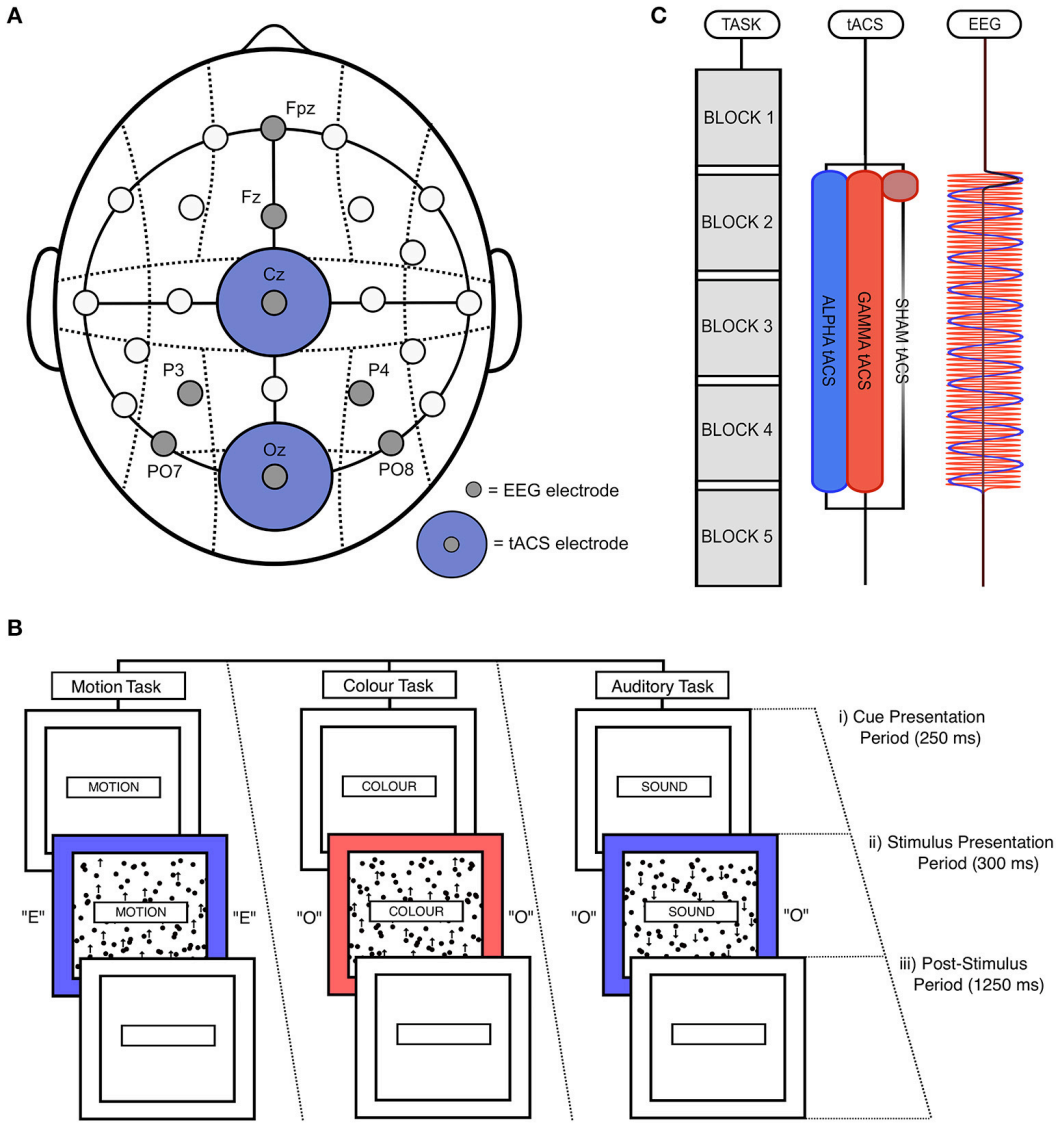
Experimental design. **(A)** tACS and EEG electrode positioning. EEG electrodes were positioned at PO7, PO8, P3, P4, Fz, and Fpz. tACS electrodes were positioned at Oz and Cz. **(B)** Illustration of a single, audio-visual switch trial. **(i)** During the cue presentation period, which lasted for 250 ms, a word was presented in the central box telling participants which task to perform (“MOTION,” “COLOUR,” or “SOUND”). **(ii)** This was followed by the stimulation presentation period, during which the outer box filled with either red or blue, the inner box filled with black dots that moved in either an upward or downwards direction, and the letter “E” or “O” was played through earphones. This period lasted for 300 ms. **(iii)** Participants could respond until the end of the post-stimulus period, which lasted for 1,250 ms. **(C)** Task and stimulation timing. Participants performed five task blocks in a single task session, with each block lasting 4 min and 50 s. A fixed-duration break of 100 s was allowed between blocks. EEG was recorded before and after the delivery of stimulation. During alpha and gamma tACS, stimulation was applied for 19 min and 30 s from the start of the second block to the start of the fifth block. During sham tACS, stimulation was applied at 10 Hz during only the first 50 s of this period (including ramp-up and down times). Participants each performed two task sessions (i.e., 8 blocks in total), separated by a break of 25 min.

EEG data were recorded using a *Starstim*® device (Neuroelectrics, Barcelona) with Ag/AgCl coated electrodes (diameter = 12 mm, contact area = 1 cm^2^). These electrodes were placed at PO7, PO8, P3, P4, Fz, and FPz. Two electrodes (*Covidien*, H124SG) were positioned on and just below the right mastoid bone. tACS was delivered using the same *Starstim*® device through two 25 cm^2^ circular sponge electrodes placed at Oz and Cz (Figure 1A). This tACS montage has been found to enhance posterior alpha power when applied at 10 Hz (Zaehle et al., 2010; Helfrich et al., 2014; Vossen et al., 2015). Modeling studies also suggest that this tACS montage directs current flow through occipitoparietal cortex (Neuling et al., 2012b; Vosskuhl et al., 2015). All electrodes were positioned using a *Neuroelectrics*® cap according to the 10–20 system. tACS electrodes were soaked in saline solution and coated with conductive electrolyte gel (*Signagel*®, Parker Laboratories) to ensure good conductivity with the scalp. EEG electrodes were filled with the same conductive gel. Stimulation was delivered at a maximum intensity of 2 mA (peak-to-peak). However, for subjects who found this intensity too unpleasant or distracting (*n* = 10), stimulation amplitudes were lowered to ensure that subjects were comfortable throughout. This approach of using variable stimulation intensities is well established in the field (e.g., Neuling et al., 2013, 2015; Kasten et al., 2016). Mean stimulation intensity was therefore 1.81 mA (*SD* = 0.35). Participants who could not tolerate <1 mA stimulation did not participate in the experiment and were replaced (*n* = 2). To ensure that the subjective effects of stimulation (e.g., scalp sessions and phosphenes) did not differ between our stimulation conditions, participants were asked at the end of the experiment to say in which task session they thought these subjective effects were most intense. Only 55% said that the subjective effects of stimulation were more intense during the alpha tACS session. A binomial test indicated that this proportion was not significantly greater than chance (i.e., 50%; *p* = 0.636). We therefore concluded that the subjective effects of stimulation did not differ reliably between alpha and control tACS in this experiment.

### Audiovisual switching task

All stimuli were presented on a Dell® 23-inch LCD monitor (60 Hz refresh rate) using the Psychophysics Toolbox in MATLAB (Brainard, 1997). The timing of stimulation and EEG recording was controlled using *MatNIC*—a toolbox designed by *Neuroelectrics*® to enable control of tACS and EEG using MATLAB. Throughout the task, a central box with a black outline was presented in the center of a gray screen (“cue box”; [height = 15 cm; width = 1.8 cm]). Two larger boxes were presented behind this cue box, the smaller of which would contain moving dots (“motion box”; [height = 12.5 cm; width = 10 cm]), and the larger of which would fill with color during stimulus presentation (“color box”; [height = 15 cm; width = 13.5 cm]). At the start of each trial, a single word (RGB = [0, 0, 0]) was displayed in the center of the cue box for 250 ms (known as the “cue presentation period”; Figure 1Bi). These cues told participants which task they would need to perform in the upcoming trial (either “MOTION,” “COLOUR,” or “SOUND”). Following this cue presentation, participants were simultaneously presented with motion, color, and auditory stimuli for a period of 300 ms (“stimulus presentation period”; Figure 1Bii). During this time, the color box turned either red (RGB = [255, 100, 100]) or blue (RGB = [100, 100, 255]). During the same period, a grid of black dots (diameter = 0.5 cm) was presented inside the motion box, which moved with 100% coherence in either an upward or downward direction (at a rate of 50 pixels per second). No dots were presented outside of the motion box. In addition, participants were also played audio, through earphones, of the vocalization of the letters “E” or “O.” These audio files were downloaded from the sound archive of the Psychology Experiment Building Language (http://prdownloads.sourceforge.net/pebl/pebl-sounds-0.1.zip?download). If participants had been cued to perform the motion task, they needed to determine whether the central dots moved in an upward or downward direction. If participants had been cued to perform the color task, they were required to classify the shade of the outer box as either red or blue. Lastly, if participants had been cued to perform the auditory task, they had to classify the auditory vocalization as either “E” or “O.” Response keys were always “M” and “Z” on the keyboard. Stickers were placed on these keys to aid performance.

Following the stimulus presentation period (“post-stimulus period”; Figure 1Biii), the cue, motion, and color boxes filled with blank, white space. This post-stimulus period lasted for 1,250 ms. Responses were recorded and timed from the beginning of the stimulus presentation period. However, the majority of responses occurred during the post-stimulus period. If a response was not recorded during this period, the trial was classified as a missed trial. The response profiles of all tasks was pseudo-randomized such that uncued tasks signaled the same response as the cued task on 50% of trials, and signaled the opposite response on the other 50% of trials. For example, if participants were cued to perform the motion task, and the correct response for this task was a left button press, the simultaneously presented auditory and color stimuli would signal the same response of 50% of trials. This was done to control for effects of inter-task response congruency.

### General experimental design

Subjects completed a first practice session, which lasted for approximately 6 min. This allowed them to familiarize themselves with the rules of the task. During this period, participants performed each task individually (motion, color, auditory) in separate blocks of 60 trials. Participants then completed a full block of the main task (162 trials), in which they were required to switch between tasks when cued. Following this first practice, EEG and tACS electrodes were positioned on the head. Once the set up was complete, participants then performed a second practice session in which they again completed a full block of the main task. Feedback was given after every trial in all practice sessions, with words “CORRECT” (Green; RGB = [100, 255, 100]) and “ERROR” (Red; RGB = [255, 100, 100]) presented below the color box following correct vs. error trials, respectively.

In the main experiment, subjects completed 2 sessions of 5 task blocks, with each block consisting of 162 trials and lasting 4 min and 50 s. Participants were given a fixed-duration break of 100 s between blocks. During this rest period, participants were shown images of pleasant, natural scenes, which changed every 20 s. These images were chosen to facilitate recovery from mental fatigue (e.g., Kaplan, 1995). In total, each session lasted 30 min and 50 s. All participants received alpha (10 Hz) tACS in one task session and either sham or gamma (50 Hz) tACS (control tACS) in the other session. Participants were randomly assigned to the sham and gamma control groups, and the order of alpha and control tACS was counterbalanced across subjects. Both the experimenter and subjects were blinded to stimulation condition. During alpha and gamma tACS, stimulation was applied for 19 min and 30 s from the start of the second block to the start of the fifth block. Sham tACS was applied at 10 Hz during only the first 50 s of this period. This sham stimulation was ramped up over 30 s and ramped down over 20 s (Figure 1C). Alpha and control tACS sessions were separated by a break of 25 min in which participants watched a nature documentary. Participants were told to relax during this period.

On one third of trials (i.e., ~33%), participants performed the same task as in the previous trial (i.e., repeat trials). On the other two thirds of trials (i.e., ~66%), participants performed a different task to the previous trial (i.e. switch trials). Each switch type (e.g., motion-color, color-motion, auditory-color, etc.) occurred with equal frequency. Participants therefore switched task on 108 trials, and each switch type was performed 18 times per block. Tasks were also performed with equal frequency (54 trials in each block).

### Behavioral analyses

All data analyses were performed using MATLAB. Mean percentage accuracy and median reaction times (RTs) were calculated for all trial types. Analyses were focused on visuovisual (within-modality) and audiovisual (cross-modality) switch trials. Visuovisual switch trials were defined as visual trials performed following a different visual task (i.e., color-motion, motion-color). Audiovisual switch trials were defined as visual trials performed following the auditory task (i.e., auditory-motion, auditory-color). These data were then submitted, separately for accuracy and RTs, to a mixed, repeated measures ANOVA with within-subjects factors of “stimulation type” (alpha vs. control tACS), “previous task modality” (visual vs. auditory), “current task type” (motion vs. color), and “task block” (2–5). Performance data from block 1 was excluded from analysis to allow us to focus on the effects of tACS (which was delivered in blocks 2–4). However, our results did not change significantly when all tasks blocks were included. We also assessed the effects of alpha tACS on visuoauditory switching as a secondary analysis (i.e., motion-auditory and color-auditory trials). We performed this analysis by taking visuoauditory switch performance during alpha vs. control tACS sessions (both accuracy and RTs), and comparing it to mean performance on visuovisual and audiovisual switch trials in each of these sessions. In all analyses, stimulation order and control group were included as between-subjects factors. Where there were violations of the assumption of sphericity, the Huynh-Feldt correction was applied. In these cases, the corresponding epsilon value (ε) is stated alongside the ANOVA results.

### EEG analyses

The first step of our EEG analysis was to divide our EEG data into multiple, 1-s segments. Where the difference between the lowest and highest value of a segment was greater than 150 μV, that segment was excluded from further analyses. Bad channels were identified manually and excluded. Power spectra were then calculated for each of these segments using the “ft_freqanalysis” function of the Fieldtrip toolbox (Oostenveld et al., 2010). Power was estimated for frequencies between 1 and 40 Hz, with a frequency resolution of 0.5 Hz. Multi-tapering, using discrete prolate spheroidal sequences, was applied with 1 Hz spectral smoothing. To determine individualized alpha bands, average power spectra for each subject were calculated from EEG data collected during both task sessions from all posterior electrodes (PO7, PO8, P3, P4). Individual alpha peak frequencies (IAFs) were then identified by manually picking the largest peak in the spectrum within an extended alpha band of 6–14 Hz (Haegens et al., 2014). For participants with identifiable alpha peaks, the alpha band was defined as IAF ± 2 Hz (e.g., Klimesch et al., 1996; Franciotti et al., 2011). For participants displaying no identifiable alpha peaks (7.9%), a canonical alpha band of 8 – 12 Hz was used. The significance of results and effect sizes were unaffected when these participants were excluded from analyses. We also observed no significant interactions when the presence of alpha peaks was included as a between-subject factor (identifiable vs. unidentifiable).

To determine the effect of tACS on EEG alpha power, we calculated power spectra for the 4 min and 50 s of EEG data recorded during the task blocks before and after stimulation for each subject. Using the same approach as Neuling et al. (2013), post-stimulation power spectra were divided by pre-stimulation power spectra to produce a measure of percentage power change for each frequency band, for each subject, in each task session. These percentage change values were then submitted to a two-way repeated-measures ANOVA with the within-subjects factors of “stimulation type” (alpha vs. control tACS) and “frequency band” (individualized theta, alpha, and low beta). Again, where there were violations of the assumption of sphericity, the Huynh-Feldt correction was applied. Individualized theta bands were defined as IAF-6 – IAF-2 Hz. Individualized beta bands were defined as IAF+2 − IAF+6 Hz. As with our behavioral analyses, stimulation order and control group were included as between-subjects factors.

### Behavioral-EEG regression analyses

We also assessed the association between the behavioral and electrophysiological effects of alpha tACS. To do this, we first subtracted RTs and percentage accuracy for visuovisual switches from those of audiovisual switches performed during and after the delivery of alpha vs. control tACS. This gave us a single measure of the effect of alpha tACS on visuovisual vs. audiovisual switching for each subject. We then generated a single measure of the effect of alpha tACS on EEG by subtracting normalized percentage change in alpha power following alpha vs. control tACS. We ran a linear regression analysis using these measures (separately for RT and percentage accuracy data).

## Results

### Planned analyses

#### Task accuracy

**Figure 2:**
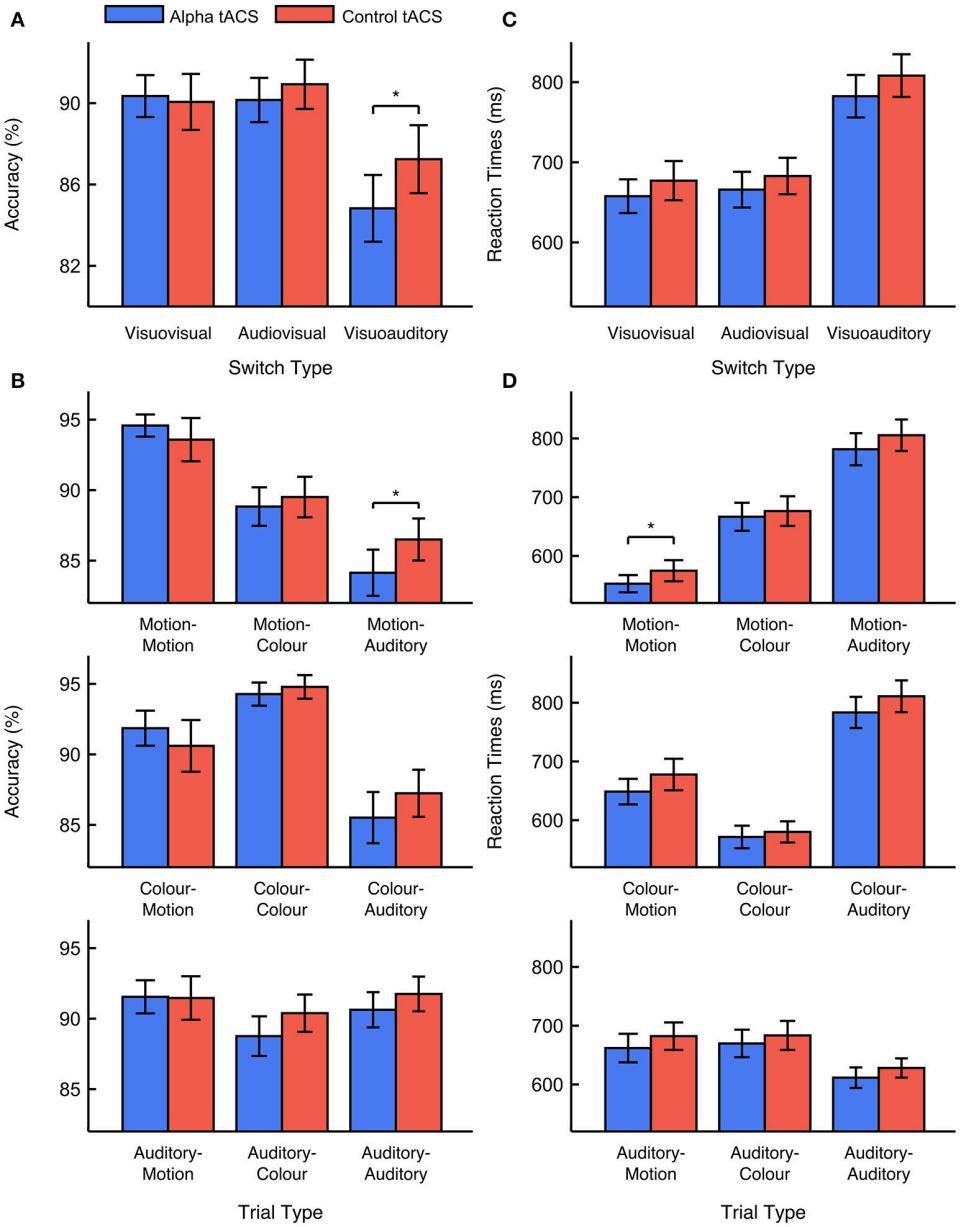
Effects of alpha vs. control tACS on task performance. **(A)** Mean performance accuracy for visuovisual, audiovisual, and visuoauditory switch trials. In contrast to predictions, alpha tACS had no effect on visuovisual vs. audiovisual switching, but did impair accuracy on visuoauditory switch trials. **(B)** Mean performance accuracy is displayed for all trial types. **(C)** Median RTs for visuovisual, audiovisual, and visuoauditory switch trials**. (D)** Median RTs are displayed for all trial types. Error bars show ±1 standard error of the mean. ^*^ = *p* < 0.05.

We focused first on task accuracy. We observed no main effects of stimulation type or previous task modality [*F*_(1, 33)_ < 1, ANOVA]. Although we predicted that alpha tACS would impair visuovisual switching specifically, we also observed no interaction between stimulation type and previous task modality [*F*_(1, 33)_ = 1.687, *p* = 0.203, $\eta_{p}^{2}$ = 0.049, ANOVA]. This suggests that alpha tACS had no reliable effect on visuovisual vs. audiovisual switching accuracy. Nevertheless, supplementary analysis did show a trend for task accuracy to be lower during alpha vs. control tACS sessions on visuoauditory switch trials [i.e. vs. visuovisual and audiovisual switches; *F*_(1, 33)_ = 3.501, *p* = 0.070, $\eta_{p}^{2}$ = 0.096, ANOVA]. This suggests that alpha tACS might impair switching away from visual tasks, toward the auditory task. We observed no higher-order interactions with stimulation order, suggesting that the effects of stimulation did not depend on whether alpha tACS was delivered in the first or second task session [*F*_(1, 33)_ < 1, ANOVA]. We also observed no significant interactions with control type [*F*_(1, 33)_ < 1, ANOVA], suggesting that the effects of sham and gamma tACS did not differ reliably from each other. We therefore concluded that, although alpha tACS exerted a trend-level effect on visuoauditory switching accuracy, this stimulation did not impair visuovisual vs. audiovisual switching accuracy in the way we anticipated. Mean percentage accuracy values for visuovisual, audiovisual, and visuoauditory switch trials during alpha vs. control tACS are displayed in Figure 2A. Mean accuracy values for all trial types during alpha vs. control tACS are displayed in Figure 2B.

#### Reaction times

We looked next at RTs. Here, we observed a marginal, main effect of stimulation type [*F*_(1, 33)_ = 2.974, *p* = 0.094, $\eta_{p}^{2}$ = 0.083, ANOVA]. This effect was driven by faster RTs during alpha vs. control tACS sessions (663 vs. 682 ms). We also observed a marginal, main effect of previous task modality [*F*_(1, 33)_ = 3.350, *p* = 0.076, $\eta_{p}^{2}$ = 0.092, ANOVA]. This effect was driven by faster RTs on visuovisual vs. audiovisual switch trials (669 vs. 676 ms). However, the important interaction between stimulation type and previous task modality was not found to be significant [*F*_(1, 33)_ < 1, ANOVA]. This suggests that alpha tACS exerted no reliable effect on visuovisual vs. audiovisual switch trial RTs. Secondary analysis also revealed no effect of alpha tACS on visuoauditory switching [i.e., vs. visuovisual and audiovisual switches*; F*_(1, 33)_ < 1, ANOVA]. We again observed no higher order interactions with either stimulation order or control type [*F*_(1, 33)_ < 1, ANOVA]. The former result suggests that the effects of stimulation on RTs did not differ depending on whether alpha tACS was delivered in the first or second task session [*F*_(1, 33)_ < 1, ANOVA]. The latter result suggests that the effects of sham and gamma tACS were approximately equivalent. We therefore concluded that, although alpha tACS seemed to exert an overall, enhancing effect on RTs, it did not influence visuovisual vs. audiovisual switch RTs in the manner we expected. Mean RT values for visuovisual, audiovisual, and visuoauditory switches during alpha vs. control tACS are displayed in Figure 2C. Mean RT values for all trial types during alpha vs. control tACS are displayed in Figure 2D.

#### EEG analyses

**Figure 3:**
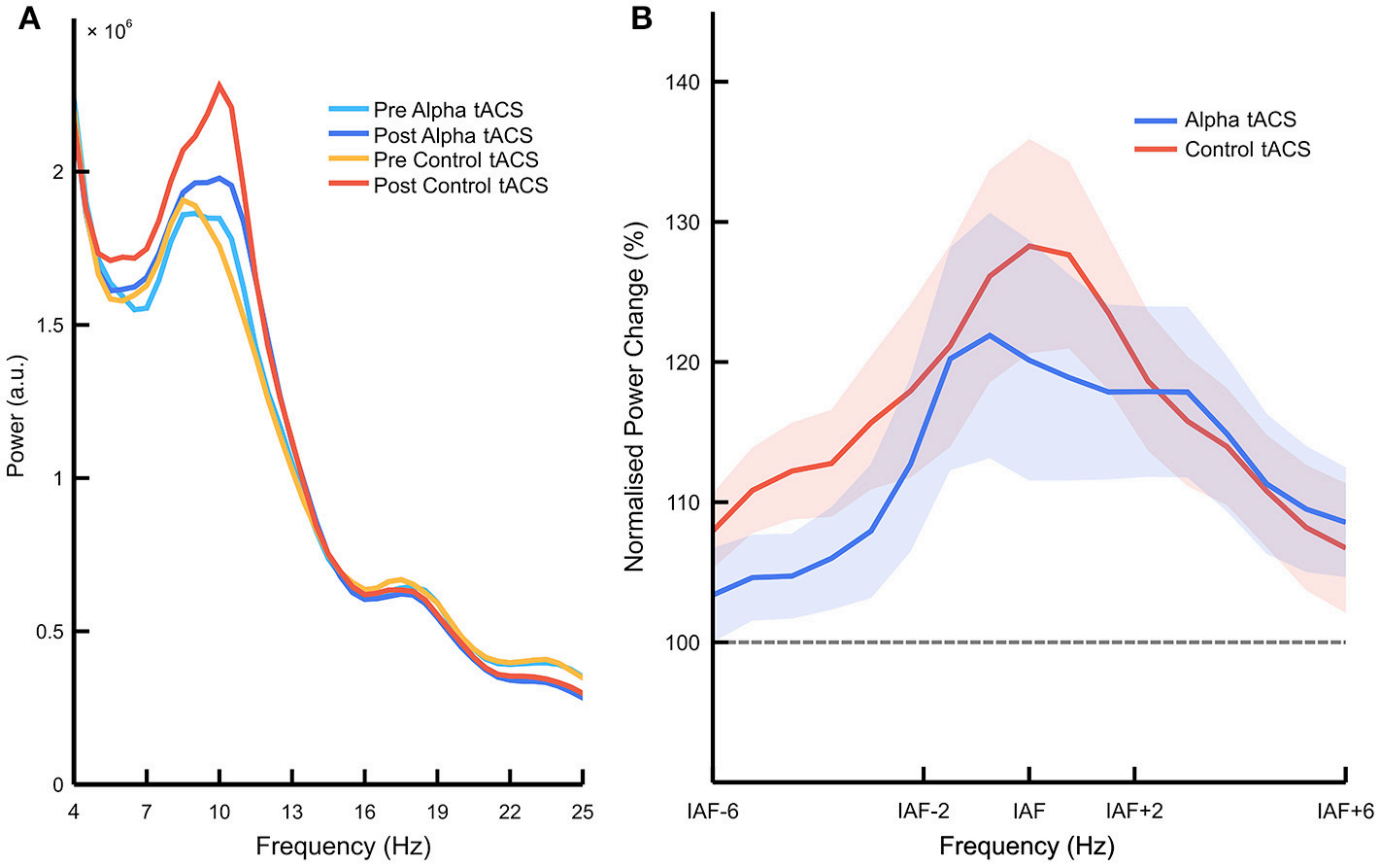
Effects of alpha vs. control tACS on overall alpha power. **(A)** Raw EEG power spectra. Mean power is plotted between 4 and 25 Hz, averaged over posterior electrodes (i.e., PO7, PO8, P3, and P4) before vs. after the delivery of alpha vs. control tACS. **(B)** Normalized percentage change in EEG power. Relative changes in posterior EEG power (i.e., post-tACS/pre-tACS) are plotted for alpha vs. control tACS. Individualized alpha bands were defined as 2 Hz above and below individualized alpha frequency (IAF). Alpha tACS was not found to exert an influence on EEG alpha power beyond that of control tACS. Shading shows ±1 standard error of the mean.

Following these behavioral analyses, we looked at tACS-related changes in EEG power. Figure 3A shows raw frequency power spectra before vs. after the delivery of alpha vs. control tACS (see Supplementary Figure 1 for individual spectra for each participant). However, our main analysis compared normalized percentage change in EEG power between stimulation conditions. We observed a significant main effect of frequency band [*F*_(2, 66)_ = 8.647, *p* < 0.001, $\eta_{p}^{2}$ = 0.208, ANOVA], indicating that power increased from the start to the end of each task session more consistently in the alpha band than in both the theta and beta bands [*M* = 119.3 vs. 106.4%, *SD* = 26.6%, *t*_(36)_ = 2.955, *p* = 0.005, *d* = 0.49, paired-samples *t*-test]. This observation replicates typical findings in the field showing increases in EEG alpha power with increased mental fatigue (e.g., Lim et al., 2013; Wascher et al., 2014). However, the important interaction between stimulation type and frequency band was not significant [*F*_(2, 66)_ = 1.695, *p* = 0.191, $\eta_{p}^{2}$ = 0.049, ANOVA]. While this indicates that alpha tACS had no specific effect on EEG alpha power, we did observe a significant interaction between stimulation type, frequency band, and stimulation order [*F*_(2, 66)_ = 10.737, *p* < 0.001, $\eta_{p}^{2}$ = 0.245, ANOVA]. Decomposition of this interaction revealed that, for EEG data collected during the first task session, there was a main effect of frequency band [*F*_(2, 70)_ = 9.759, *p* < 0.001, $\eta_{p}^{2}$ = 0.218, ANOVA], but no effect of stimulation [*F*_(1, 35)_ < 1, ANOVA], and no interaction between stimulation type and frequency band [*F*_(2, 70)_ < 1, ANOVA]. For EEG data collected during the second task session, we observed no main effect of either frequency band [*F*_(2, 70)_ < 1, ANOVA] or stimulation [*F*_(1, 35)_ < 1, ANOVA], and no interaction between stimulation type and frequency band [*F*_(2, 70)_ < 1, ANOVA]. This suggests that alpha power increased more significantly during the first vs. second task session (vs. theta + beta; *M* = 12.6%, *SD* = 24.0%, *t*_(18)_ = 2.294, *p* = 0.034, *d* = 0.53, paired-samples *t*-test), but that these changes in alpha power were not influenced by the delivery of alpha vs. control tACS in either session (Supplementary Figure 2). We observed no such higher order interactions with control group (*p* > 0.2), suggesting that effects of sham and gamma tACS were approximately equivalent. We therefore concluded that, despite previous results showing enhancements of EEG alpha power following alpha tACS (e.g., Helfrich et al., 2014; Neuling et al., 2015), we did not observe reliable enhancement of this kind in the current experiment (Figure 3B). Percentage changes in IAF-centered power are plotted for each participant in Supplementary Figure 3.

#### Behavioral-EEG correlations

In the last of our planned analyses, we looked at associations between the behavioral and electrophysiological effects of alpha tACS. To do this, we compared a single measure of percentage change in alpha power following alpha vs. control tACS with a single measure of the effect of alpha tACS on visuovisual vs. audiovisual switching accuracy and RTs. However, this individual difference correlation revealed no significant association between changes in EEG alpha power and either task accuracy [β = −0.147, *F*_(1, 36)_ < 1, linear regression] or RTs [β = −0.105, *F*_(1, 36)_ < 1, linear regression). We therefore concluded that there were no associations across participants between alpha tACS related changes in EEG alpha power and visuovisual vs. audiovisual task switching.

### Exploratory analyses

#### Behavioral analyses

As described above, the results of our planned analysis did not support our initial predictions. In fact, these analyses suggested that alpha tACS had little effect on either EEG or task performance. In response to these results, we next sought to perform exploratory, *post-hoc* analyses to investigate the presence of unanticipated patterns in our data.

We first assessed whether alpha tACS had an effect on RT variability. RT variability has previously been identified as a measure of task performance that can provide unique insights into attentional processes beyond those of accuracy and RT central tendencies (e.g., Esterman et al., 2013). To conduct this analysis, RT variability measures were submitted to the same repeated measures ANOVA as median RTs. However, these results were not significantly different to those of median RTs (Supplementary Figure 4A). There were no main effects of stimulation type or previous task modality (*p* > 0.3). There were also no interactions between stimulation type and either previous task modality or current task type (*p* > 0.3). This suggests that alpha tACS exerted no influence on visuovisual vs. audiovisual switching with respect to RT variability.

We then analyzed our behavioral data using a diffusion model. We did this to allow measurement of task performance, while considering task accuracy and RTs simultaneously. This enabled us to assess task performance using measures that are less sensitive to speed-accuracy trade-offs. This was important given that alpha tACS was found to reduce overall RTs, indicating a possible trend toward impulsive responding. To perform this analysis, we used the EZ-diffusion model proposed by Wagenmakers et al. (2007). Using this model, the following measures were calculated from mean RTs, RT variability, and task accuracy: (1) *drift rate*, which estimates the quality (or signal-to-noise ratio) of information processing, (2) *boundary separation*, which estimates participant bias toward responding and (3) *non-decision time*, which estimates the time taken for participants to process stimuli and respond. We did observe a marginal, main effect of stimulation type on non-decision times [*F*_(1, 33)_ = 4.092, *p* = 0.051, $\eta_{p}^{2}$ = 0.110, ANOVA], driven by reduced non-decision times on visuovisual vs. audiovisual switch trials (433 vs. 450 ms). However, for all other measures, we observed no significant main effects or higher-level interactions. Consequently, using diffusion model parameters that are less sensitive to speed-accuracy trade-offs, we concluded that, with the exception of a possible influence of alpha tACS on stimulus processing times, this stimulation had no effect on visuovisual vs. audiovisual task switching (Supplementary Figures 4B–D).

We also assessed whether alpha tACS had an effect on trials where participants performed the same task twice (“repeat trials”). We did this by calculating mean accuracy and RTs for repeat trials in the four blocks that followed the beginning of alpha vs. control tACS (i.e., blocks 2–5). We then submitted this data to a 3-way repeated measure ANOVA, with within-subjects factors of stimulation type, task type (motion, color, auditory), and task block. However, we again observed no effects in either accuracy or RTs beyond those of our switch analyses (*p* > 0.3) (Supplementary Figures 5A,B). Lastly, we assessed whether the effects on alpha tACS on visuovisual switching were affected by individual differences in task difficulty. This question was partly inspired by recent reports that differences between individuals in their aptitudes for given tasks can affect the direction with which transcranial electrical stimulation influences their task performance (Sarkar et al., 2014; Looi et al., 2016; Popescu et al., 2016). Specifically, we asked whether alpha tACS might have exerted different effects on switching between visual tasks that participants found easier vs. more difficult, compared to switching from the difficult to easy task. To conduct this analysis, we looked at median RTs for each subject on motion vs. color trials. The task with faster RTs was defined as the “easier” task for that subject, while the other task was defined as “harder.” We then calculated mean accuracy and RTs for “Easy-Hard” and “Hard-Easy” switch trials for alpha vs. control tACS sessions. This reformatted data was then submitted to a two-way repeated-measures ANOVA, with within-subjects factors of “trial type” and “stimulation type.” We observed significant main effects of trial type in both accuracy [*F*_(1, 33)_ = 5.154, *p* = 0.030, $\eta_{p}^{2}$ = 0.135, ANOVA] and RTs [*F*_(1, 33)_ = 15.865, *p* < 0.001, $\eta_{p}^{2}$ = 0.325, ANOVA]. This reflects the unsurprising fact that performance was slower and less accurate when participants switched to the harder task. However, we observed no other effects beyond those previously reported (*p* > 0.2) (Supplementary Figures 5C,D). We therefore found no evidence in either accuracy or RTs to support the idea that individual differences in task difficulty influenced the effects of alpha tACS on visual task switching.

#### EEG analyses

In addition to these behavioral analyses, we also ran *post-hoc* analyses of our EEG results. Given that alpha tACS exerted no effects on endogenous alpha power (i.e., alpha measured within a 5 min period before vs. after tACS delivery), we wondered whether alpha tACS might instead have influenced stimulus-evoked changes in alpha power. In general, significant reductions in alpha power are observed following presentation of visual stimuli. This process is known as event-related desynchronisation (ERD; Pfurtscheller and Da Silva, 1999; Li et al., 2013). Importantly, in a recent study by Kasten and Herrmann (2017), alpha tACS was found to enhance the amplitude of ERD. We therefore investigated whether similar effects were present in our data.

To perform this analysis, we took EEG data recorded from posterior electrodes (PO7, PO8, P3, and P4) before and after the delivery of alpha vs. control tACS. After filtering this data between IAF ± 2 Hz, we then used the *hilbert()* function in MATLAB to extract the Hilbert transform of the data. The real component of this complex, analytic signal gave us the envelope of the filtered data and, therefore, a continuous measure of alpha power over time. To assess event-related changes in alpha power, we first extracted average, enveloped waveforms for each subject from −600 to 1,200 ms after stimulus presentations (A).We then extracted a single measure of baseline alpha power for each trial (R) by taking the mean of the enveloped signal from −600 to 0 ms. As in Kasten and Herrmann (2017), ERD was calculated using the following equation of Pfurtscheller and Da Silva (1999): ((R-A)/R)^*^100. Positive values on this measure reflect ERD, while negative values reflect event-related synchronization (see Supplementary Figure 6A for grand average across all participants). Finally, to focus on stimulus-induced changes in alpha power, mean ERD was calculated from 200 to 800 ms after stimulus presentations. These single, ERD averages were then submitted to a two-way repeated measures ANOVA with the within-subjects factors of “stimulation type” (alpha vs. control tACS) and “EEG session” (pre- vs. post-tACS).” Stimulation order and control group were again included as between-subjects factors.

Although the overall patterns of ERD were very similar in both alpha and control tACS sessions, we observed a significant interaction between stimulation type and EEG session [*F*_(1, 33)_ = 8.763, *p* = 0.006, $\eta_{p}^{2}$ = 0.210, ANOVA]. However, although this finding suggested an effect of alpha tACS on ERD, further investigation of the interaction cast doubt on its significance. For example, as shown in Supplementary Figure 6B, the interaction appeared to be driven primarily by differences in ERD occurring before the delivery of stimulation. Specifically, ERD was significantly greater before, but no different after alpha vs. control tACS. It is therefore difficult to determine whether the interaction reflects a genuine influence of alpha tACS on ERD, or simply an unanticipated difference in baseline ERD between stimulation conditions. Additionally, it should be noted that our ERD results contrast with those of Kasten and Herrmann (2017). Although these researchers observed increases in ERD following alpha tACS, our results suggest a relative reduction in ERD following alpha tACS.

## Discussion

In this pre-registered experiment, we sought to determine the effects of alpha tACS on visuovisual vs. audiovisual task switching. EEG and task performance were measured before, during, and after the delivery of alpha vs. control tACS. Previous experiments conducted by our lab suggested that alpha tACS exerts a stabilizing effect on visual attention task performance. We therefore predicted that alpha tACS would make it harder for participants to switch between visual tasks, while having little effect on audiovisual switching. However, our data did not support this prediction.

Our primary analyses revealed no effect of alpha tACS on visuovisual vs. audiovisual switching accuracy. While analysis of RT data showed that overall response times were faster during alpha vs. control tACS, the important interaction between stimulation type and previous task modality was again not found to be significant. This indicates that alpha tACS did not influence visuovisual vs. audiovisual task switching performance. However, supplementary analysis of task accuracy did indicate a mildly impairing effect of alpha tACS on visuoauditory switching. Therefore, while alpha tACS did not impair switching between visual tasks as we anticipated, this stimulation did seem to make it harder for people to switch away from visual tasks. Surprisingly, although many studies have previously reported increases in EEG alpha power following alpha tACS (Zaehle et al., 2010; Helfrich et al., 2014; Vossen et al., 2015), our EEG results also did not follow our predictions. Alpha tACS did not induce greater increases in EEG alpha power than control tACS. Furthermore, there were no associations across participants between alpha tACS related changes in EEG alpha power and visuovisual vs. audiovisual task switching. Relating to these observations, it should be emphasized that the precise methods of this experiment were not identical to those of the previously mentioned studies reporting increases in alpha power following alpha tACS. It is also notable that, although we observed no overall effects of alpha tACS on EEG power, alpha power was found to increase after 10 Hz stimulation when it was delivered in the first task session, possibly reflecting the importance of state-dependent effects of brain stimulation (Romei et al., 2016). Additionally, as our auditory task relied to a significant extent on auditory processing, it is likely that posterior alpha power in the current experiment was elevated with respect to previous studies (which often used exclusively visual tasks; e.g., Helfrich et al., 2014), due to the positive association between auditory attention and posterior alpha power (e.g., Clayton et al., 2015). Consequently, as elevated alpha power has been found to limit the enhancing influence of alpha tACS on alpha power (e.g., Neuling et al., 2012a; Ruhnau et al., 2016), this factor could also explain why the EEG results of this experiment did not match our predictions.

Following these planned comparisons, we performed a number of exploratory analyses to investigate the presence of unanticipated patterns in our data. These *post-hoc* analyses showed that alpha tACS similarly had little influence on RT variability, diffusion model parameters, or on the performance of repeat trials. The effects of alpha tACS on visuovisual switching were not affected by variation across participants in the difficulty of visual tasks. *Post-hoc* analyses of our EEG data did suggest an effect of alpha tACS on ERD following the presentation of task stimuli. However, baseline differences in ERD between stimulation conditions suggested against interpreting this effect as a genuine consequence of alpha tACS. We dedicate the following sections of this paper to the discussion of these unexpected results.

Before examining the possible reasons why alpha tACS did not influence task performance or EEG in the ways we predicted, it is important to first describe some of the general issues around transcranial electrical stimulation (tES) that may have contributed to our null results. A central challenge with tES is that it is extremely difficult to know exactly where and at what intensities stimulation has been delivered to the brain. For example, it has recently been estimated that as much as 90% of the electrical current delivered during tES travels across the skin and therefore bypasses the brain altogether (Underwood, 2016; Lafon et al., 2017). This problem of uncertainty around the delivery of electrical stimulation is compounded by the fact that, even if one could be certain about the paths of delivered electrical currents, one cannot be sure about how these electrical currents will influence brain activity. For example, due to variability in brain morphology across individuals, electrical stimulation delivered from the same scalp positions can have significantly different effects across participants on underlying cortex (Laakso et al., 2015; Opitz et al., 2015). Furthermore, the influence of tES on neural activity is not linearly associated with the intensity of stimulation, as increased excitability is often observed at intermediate intensities, but increased inhibition has been reported at higher intensities (Batsikadze et al., 2013). Lastly, even if one could know exactly where and how delivered electrical currents influenced brain activity, it would still be difficult to predict the precise, neural effects of stimulation due to the immense interconnectivity of the brain. For example, even if a given tES procedure influenced neural activity only in occipitoparietal cortex, it is highly likely that this modulation would travel across the brain, reverberating through it in an indeterminate and subject-specific manner.

It is therefore possible that we did not observe significant EEG or behavioral effects in this experiment because stimulation exerted an influence on brain activity that was more variable across participants, or substantially different in nature, to what we intended. Given that stimulation was delivered for less than 20 min in this experiment, it is also possible that a longer period of stimulation would have produced clearer effects on task performance. Nevertheless, previous experiments by our group used near-identical methods to the current study and observed replicable effects of alpha tACS on visual task performance. It is therefore important to consider the alternative possibility that, rather than alpha tACS not influencing brain activity as we intended, certain aspects of our task design may have contributed to our null results. For example, one factor that could have prevented the observation of behavioral effects during alpha tACS is the generally high levels of task accuracy in this experiment (*M* = 90.3%, *SD* = 6.4%). Although such levels do not indicate a ceiling effect, one might argue that the influence of alpha tACS on task accuracy could have been obscured by participants making very few errors overall. Nevertheless, this argument cannot be made for RTs, in which we observed no evidence of tACS-related effects on visuovisual vs. audiovisual switching. Additionally, it is also conceivable that the high probability of task switching in this experiment influenced our behavioral results. We predicted that alpha tACS would impair switching between different states of visual attention (i.e. task sets). However, it is possible that the high frequency of task switching in this experiment (66.6%) encouraged participants to engage all tasks sets concurrently (i.e., motion, color, and audition). Some participants may therefore have been near-continuously prepared to perform each task, meaning that task performance depended less on switching between task sets, and depended more on activating multiple task sets simultaneously. Evidence that high switch frequencies promote continuous engagement of multiple task sets comes from findings that switch costs reduce (i.e., suggesting more effective performance of multiple tasks) both with increased switch frequency (Monsell and Mizon, 2006; Duthoo et al., 2012), and when people are uncertain about which tasks they will need to perform moment-to-moment (Lange et al., 2015). Nevertheless, despite these concerns about high switch frequency, we did observe robust switch costs in the current experiment. Consequently, if alpha tACS does influence switching between different states of visual attention, it is arguable that we should have observed reliable effects of stimulation on visuovisual vs. audiovisual switch performance.

It is clearly difficult to make conclusions from null results. Nevertheless, if we assume that alpha tACS was delivered to the brain as we intended, and that no aspect of our task design contributed to our null results, we are left with a few remaining interpretations. The first is that alpha tACS simply does not impair visuovisual task switching, and therefore may not influence the stability of visual processing. This idea is consistent with the recent finding that, while gamma tACS to posterior cortex (60 Hz) increases the rate at which people's perception of multistable images changes over time, alpha tACS exerts no such effects on the stability of ongoing, perceptual interpretations (Cabral-Calderin et al., 2015). However, it should also be noted that we did observe a trend-level, impairing effect of alpha tACS on visuoauditory task switching accuracy. Consequently, an alternative, *post-hoc* explanation for our results could be that alpha tACS does not influence switching between visual tasks, but instead exerts an impairing effect on transitions in attention away from the visual domain. As we did not predict this effect at the start of the study, further experiments will be required to investigate the replicability of such tACS-induced impairments in visuoauditory switching. However, if replicable, these impairments could perhaps explain why alpha tACS was found to support performance on sustained visual tasks in our previous experiments. Specifically, if alpha tACS impairs switching of attention away from visual tasks, this could suggest that alpha tACS helps to focus attention on ongoing, visual tasks by preventing unwanted transitions of attention away from those tasks, and toward irrelevant, non-visual processes (e.g. mind-wandering). Whatever the reasons why these specific patterns of results were observed in this study, we hope that this work demonstrates the intriguing, but highly complex effects of alpha tACS on human brain activity and behavior. We also hope this pre-registered experiment can guide the design of future experiments in the field.

## Ethics statement

This study was carried out in accordance with guidelines of the University of Oxford. Ethical approval to perform this research was given by the Clinical Trials and Research Governance (CTRG) team at Oxford, and by the Ministry of Defence Research Ethics Committee (MODREC). All subjects gave written informed consent in accordance with the Declaration of Helsinki.

## Author contributions

This study was designed by all authors. Data collection and analyses were conducted by MC. The manuscript was written by MC, with comments from RC and NY.

### Conflict of interest statement

RC serves on the scientific advisory boards of Neuroelectrics Inc. and Innosphere Inc. The authors declare that the research was conducted in the absence of any commercial or financial relationships that could be construed as a potential conflict of interest.

## Supplementary material

Supplementary Figure 1 Raw EEG power spectra for each participant. As in Figure 3A, raw power values are plotted between 4 and 25 Hz, averaged over posterior electrodes (i.e., PO7, PO8, P3, and P4) before vs. after the delivery of alpha vs. control tACS.

Supplementary Figure 2 Normalized percentage change in IAF-centered EEG power, separated by stimulation order. **(A)** Data for subjects that received alpha tACS in the first task session. **(B)** Data for subjects that received alpha tACS in the second task session. In general, alpha power increased most significantly during the first task session (i.e., during alpha tACS when alpha tACS was delivered in the first session, and during control tACS when alpha tACS was delivered in the second session). Shading shows ±1 standard error of the mean.

Supplementary Figure 3 Normalized percentage change in IAF-centered EEG power for each participant. As in Figure 3B, relative changes in posterior EEG power (i.e., post-tACS/pre-tACS) are plotted for alpha vs. control tACS.

Supplementary Figure 4 Effects of alpha vs. control tACS on **(A)** RT variance, **(B)** drift rate, **(C)** boundary separation, and **(D)** non-decision time. Error bars show ±1 standard error of the mean. ^*^ = *p* < 0.05.

Supplementary Figure 5 Effects of alpha vs. control tACS on repeat trial performance (accuracy and RTs, **A,B**), and switching between easy vs. difficult visual tasks (accuracy and RTs, **C,D**). Error bars show ±1 standard error of the mean. ^*^ = *p* < 0.05.

Supplementary Figure 6 Effects of alpha vs. control tACS on event-related desynchronisation (ERD) in IAF ±2 Hz power. **(A)** ERD waveforms averaged over posterior electrodes from −600 to 1,250 ms after stimulus presentations. The area of gray shading indicates the time window in which ERD was averaged for analysis (i.e., 200–800 ms). **(B)** Mean ERD before and after delivery of alpha vs. control tACS. A significant reduction in ERD was observed following alpha vs. control tACS. However, this difference was driven primarily by baseline differences in ERD between stimulation conditions. Error bars show ±1 standard error of the mean. ^*^ = *p* < 0.05.
