# Supplementary figures and images for: The Effects of 10 Hz Transcranial Alternating Current Stimulation on Audiovisual Task Switching
Source: Front Neurosci. 2018 Feb 13;12:67. doi: 10.3389/fnins.2018.00067 (PMC5816909; doi:10.3389/fnins.2018.00067)

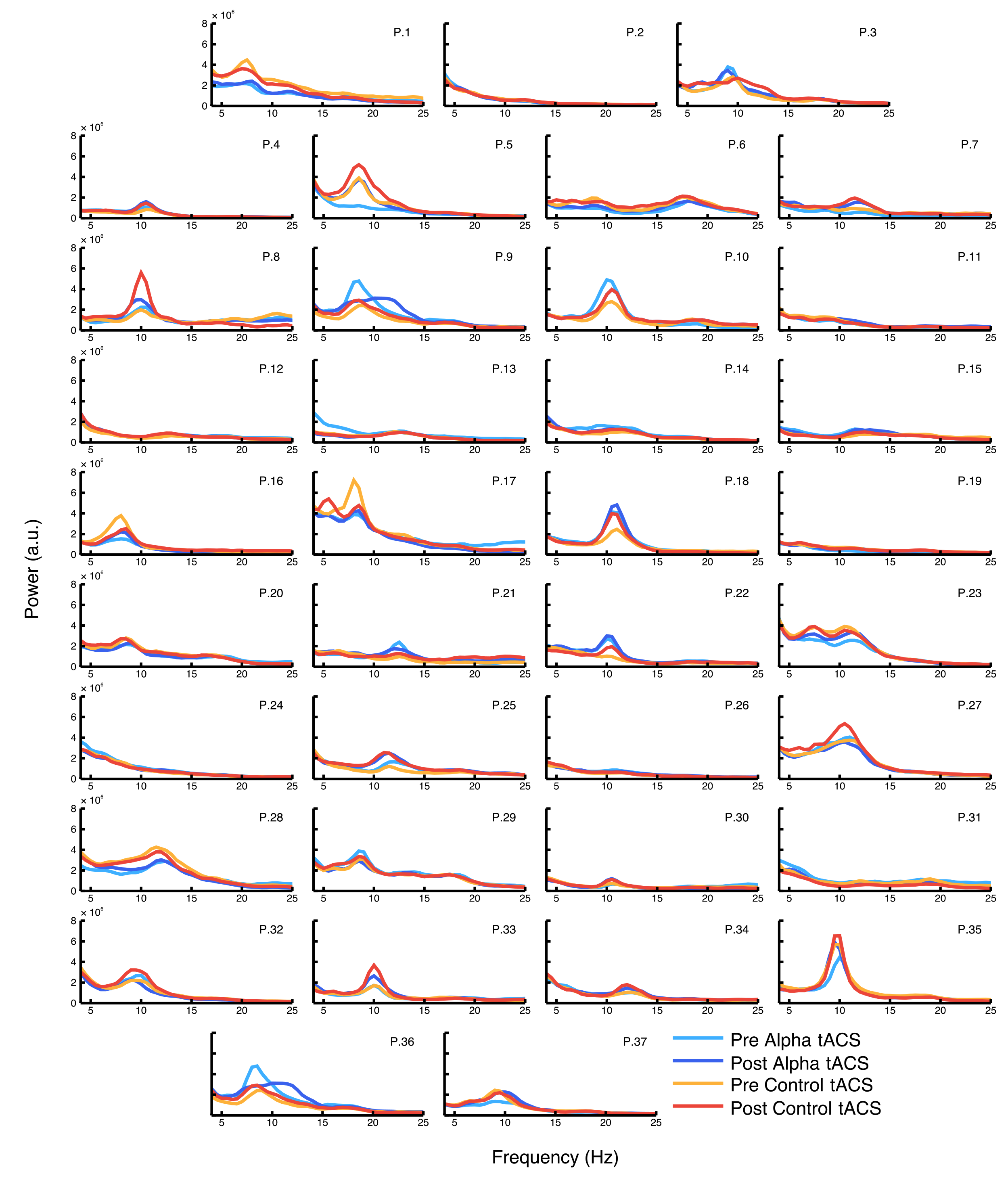

Supplement: Supplementary Figure 1 — Raw EEG power spectra for each participant. As in Figure 3A, raw power values are plotted between 4 and 25 Hz, averaged over posterior electrodes (i.e., PO7, PO8, P3, and P4) before vs. after the delivery of alpha vs. control tACS. [file Image1.TIFF]

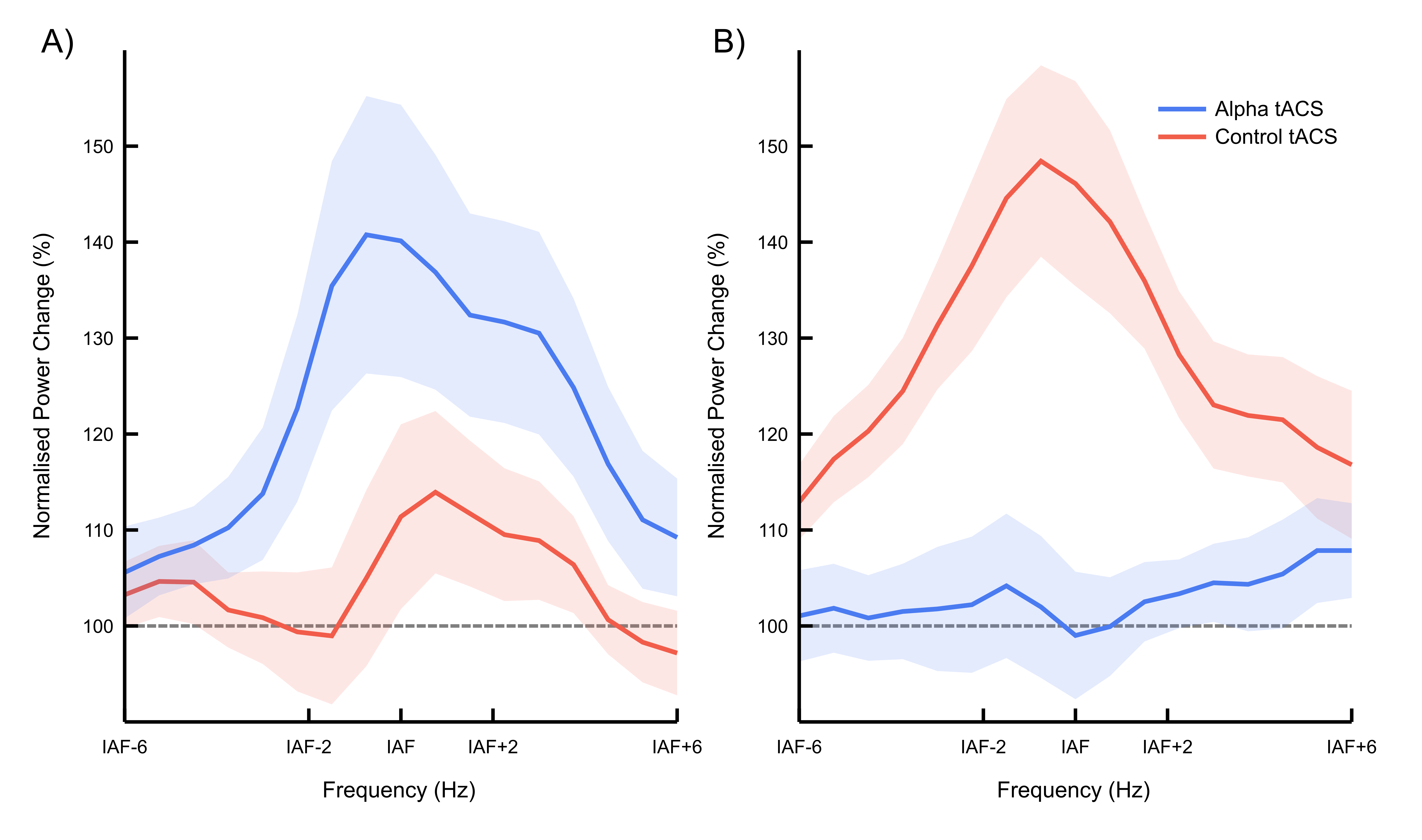

Supplement: Supplementary Figure 2 — Normalized percentage change in IAF-centered EEG power, separated by stimulation order. (A) Data for subjects that received alpha tACS in the first task session. (B) Data for subjects that received alpha tACS in the second task session. In general, alpha power increased most significantly during the first task session (i.e., during alpha tACS when alpha tACS was delivered in the first session, and during control tACS when alpha tACS was delivered in the second session). Shading shows ±1 standard error of the mean. [file Image2.TIFF]

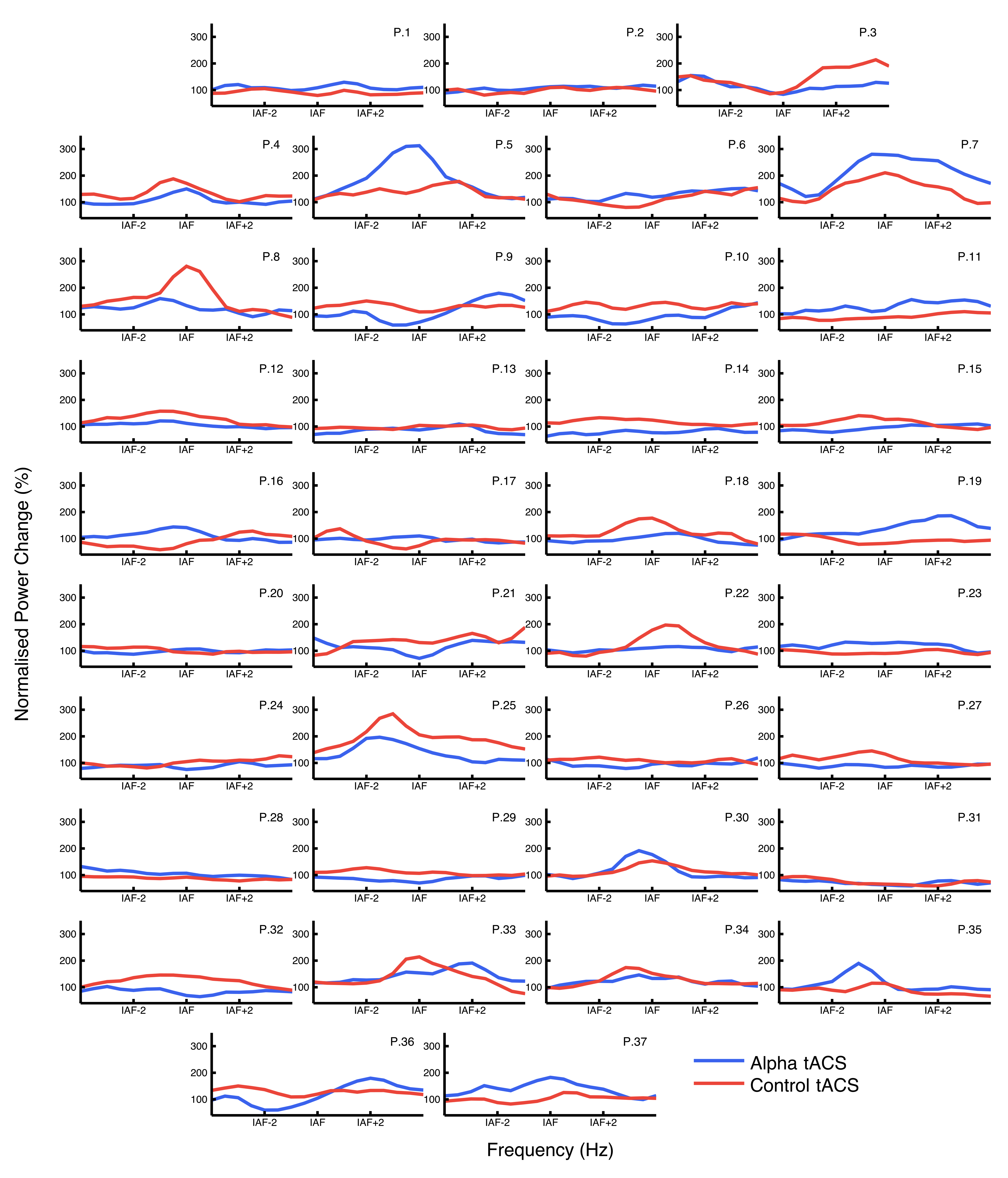

Supplement: Supplementary Figure 3 — Normalized percentage change in IAF-centered EEG power for each participant. As in Figure 3B, relative changes in posterior EEG power (i.e., post-tACS/pre-tACS) are plotted for alpha vs. control tACS. [file Image3.TIFF]

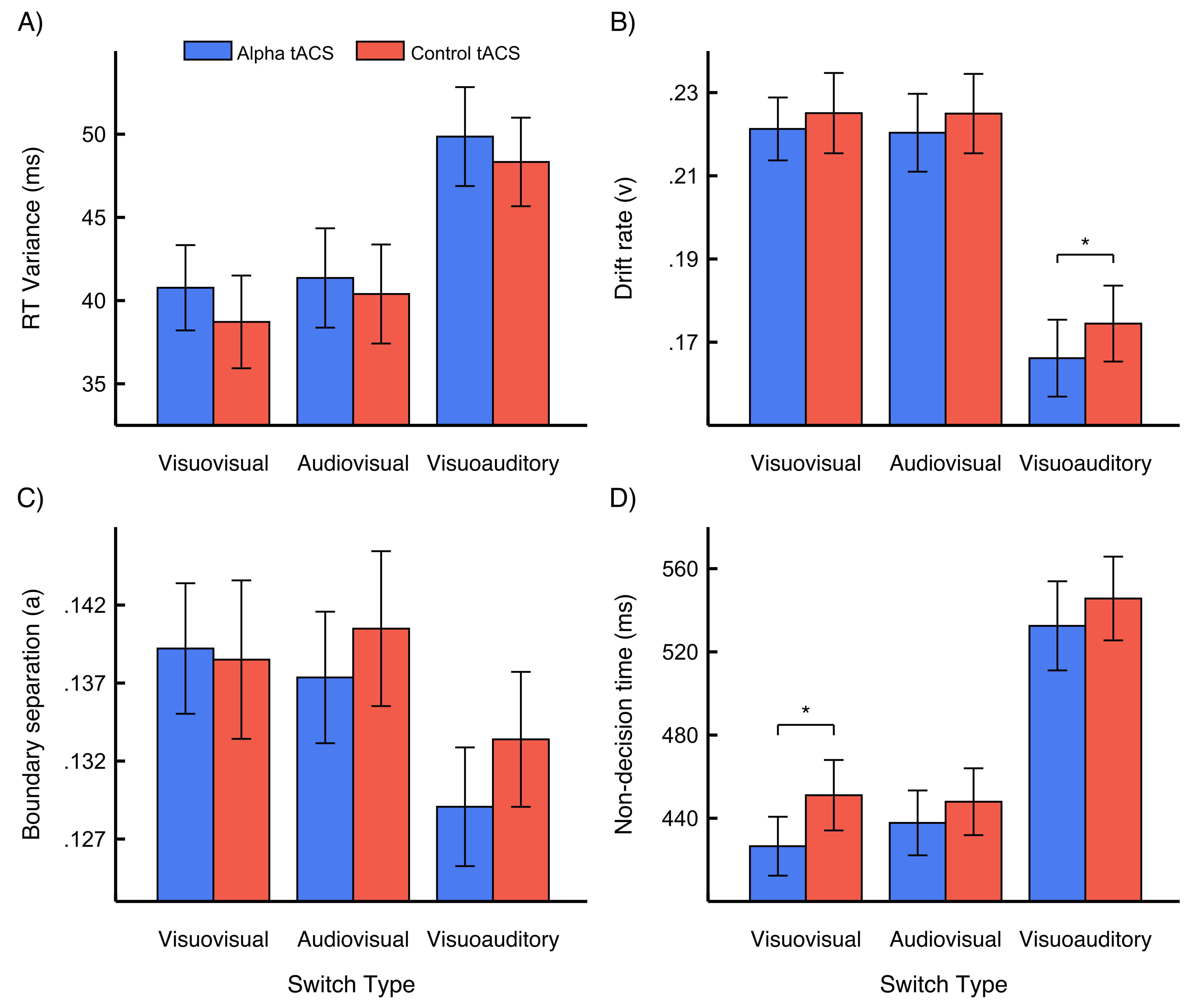

Supplement: Supplementary Figure 4 — Effects of alpha vs. control tACS on (A) RT variance, (B) drift rate, (C) boundary separation, and (D) non-decision time. Error bars show ±1 standard error of the mean. * = p < 0.05. [file Image4.TIFF]

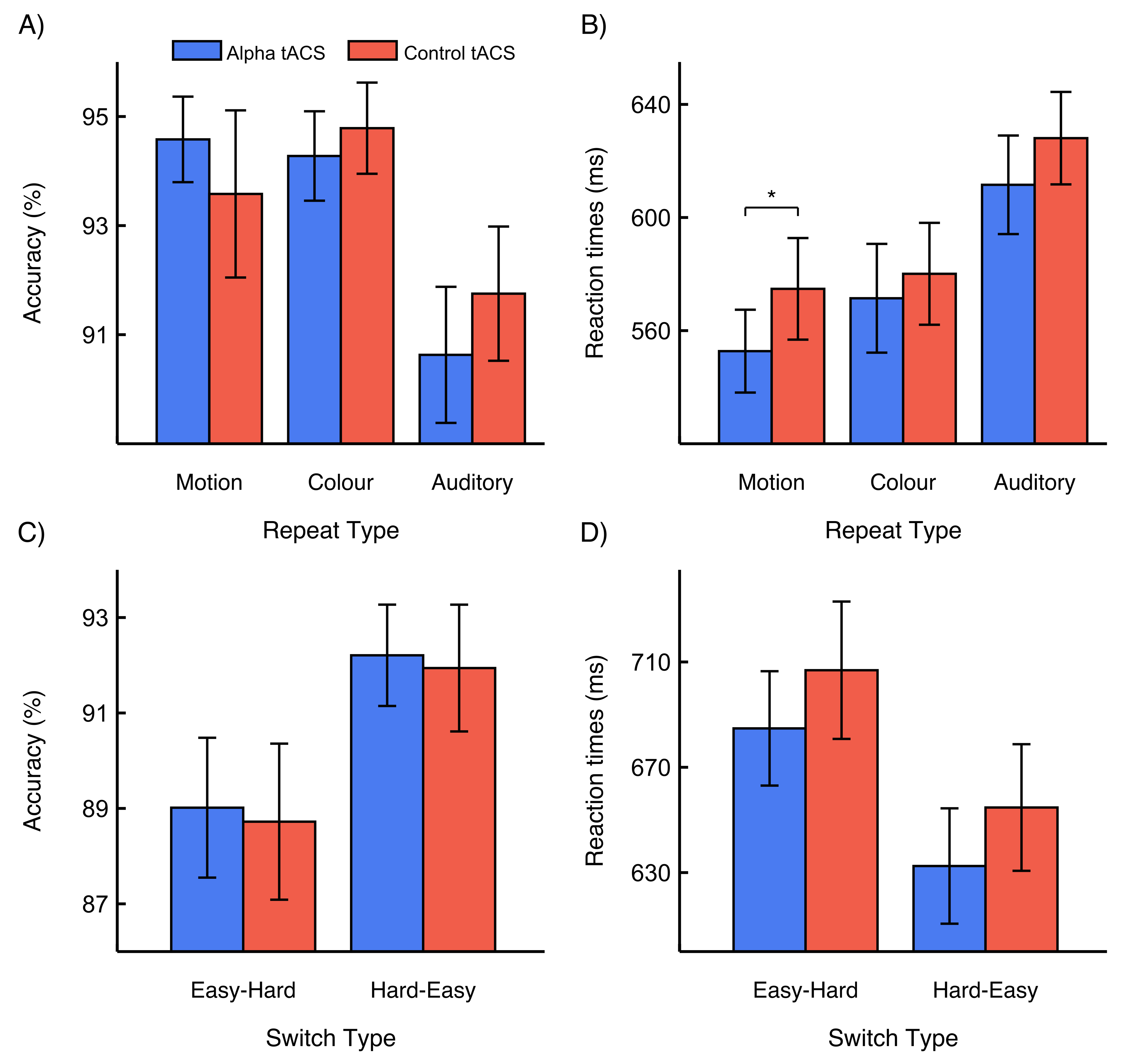

Supplement: Supplementary Figure 5 — Effects of alpha vs. control tACS on repeat trial performance (accuracy and RTs, A,B), and switching between easy vs. difficult visual tasks (accuracy and RTs, C,D). Error bars show ±1 standard error of the mean. * = p < 0.05. [file Image5.TIFF]

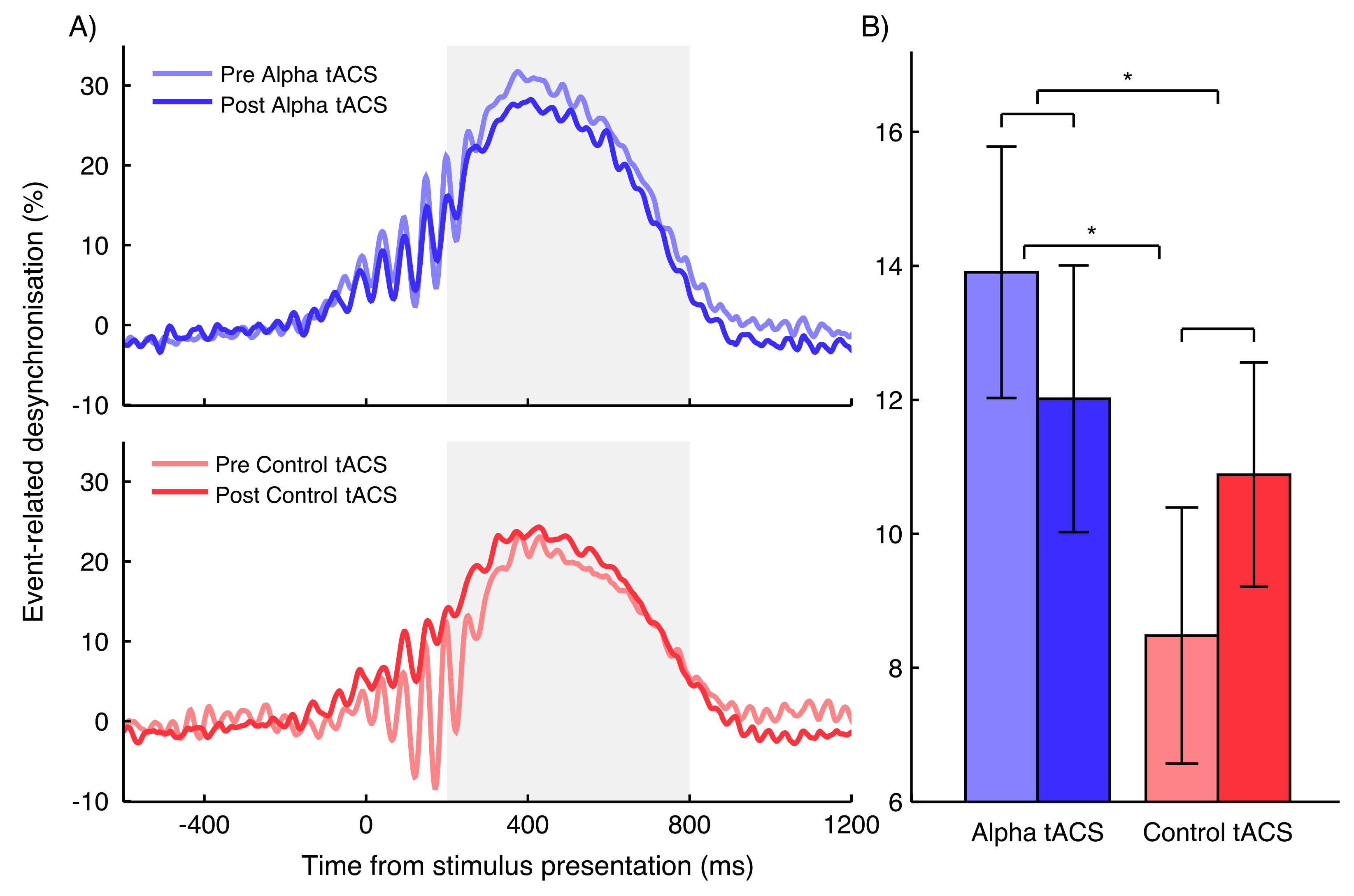

Supplement: Supplementary Figure 6 — Effects of alpha vs. control tACS on event-related desynchronisation (ERD) in IAF ±2 Hz power. (A) ERD waveforms averaged over posterior electrodes from −600 to 1,250 ms after stimulus presentations. The area of gray shading indicates the time window in which ERD was averaged for analysis (i.e., 200–800 ms). (B) Mean ERD before and after delivery of alpha vs. control tACS. A significant reduction in ERD was observed following alpha vs. control tACS. However, this difference was driven primarily by baseline differences in ERD between stimulation conditions. Error bars show ±1 standard error of the mean. * = p < 0.05. [file Image6.TIFF]
